# Characterization of anthracycline-induced cardiotoxicity by diffusion tensor magnetic resonance imaging

**DOI:** 10.1007/s00395-024-01039-z

**Published:** 2024-03-14

**Authors:** David Lohr, Arne Thiele, Max Stahnke, Vera M. Braun, Robert Klopfleisch, Oliver Klein, Sandra Dresen, Ulf Landmesser, Anna Foryst-Ludwig, Ulrich Kintscher, Laura M. Schreiber, Niklas Beyhoff

**Affiliations:** 1 Chair of Molecular and Cellular Imaging, Comprehensive Heart Failure Center (CHFC), University Hospital Wuerzburg, Wuerzburg, Germany; 2 Max Rubner Center for Cardiovascular Metabolic Renal Research, Institute of Pharmacology, Charité - Universitätsmedizin Berlin, Berlin, Germany; 3 DZHK (German Centre for Cardiovascular Research), Partner Site Berlin, Berlin, Germany; 4 Experimental and Clinical Research Center, a joint cooperation of Max-Delbrück Center for Molecular Medicine and Charité - Universitätsmedizin Berlin, Berlin, Germany; 5 Department of Nephrology and Intensive Care Medicine, Charité - Universitätsmedizin Berlin, Berlin, Germany; 6 Max-Delbrück-Center for Molecular Medicine in the Helmholtz Association, Berlin, Germany; 7 Department of Veterinary Pathology, College of Veterinary Medicine, Freie Universität Berlin, Berlin, Germany; 8 Berlin-Brandenburg Center for Regenerative Therapy (BCRT), Charité - Universitätsmedizin Berlin, Berlin, Germany; 9 Berlin Institute of Health at Charité - Universitätsmedizin Berlin, Berlin, Germany; 10 Department of Cardiology, Angiology and Intensive Care Medicine, Deutsches Herzzentrum der Charité - Medical Heart Center of Charité and German Heart Institute Berlin, Berlin, Germany

**Keywords:** Anthracyclines, Cancer therapy-related cardiac dysfunction, Cardiac atrophy, Cardiotoxicity, Chemotherapy, Diffusion tensor magnetic resonance imaging

## Abstract

Anthracyclines are highly potent anti-cancer drugs, but their clinical use is limited by severe cardiotoxic side effects. The impact of anthracycline-induced cardiotoxicity (AIC) on left ventricular (LV) microarchitecture and diffusion properties remains unknown. This study sought to characterize AIC by cardiovascular magnetic resonance diffusion tensor imaging (DTI). Mice were treated with Doxorubicin (DOX; *n* = 16) for induction of AIC or saline as corresponding control (*n* = 15). Cardiac function was assessed via echocardiography at the end of the study period. Whole hearts (*n* = 8 per group) were scanned ex vivo by high-resolution DTI at 7 T. Results were correlated with histopathology and mass spectrometry imaging. Mice with AIC demonstrated systolic dysfunction (LVEF 52 ± 3% vs. 43 ± 6%, *P* < 0.001), impaired global longitudinal strain (−19.6 ± 2.0% vs. −16.6 ± 3.0%, *P* < 0.01), and cardiac atrophy (LV mass index [mg/mm], 4.3 ± 0.1 vs. 3.6 ± 0.2, *P* < 0.01). Regional sheetlet angles were significantly lower in AIC, whereas helix angle and relative helicity remained unchanged. In AIC, fractional anisotropy was increased (0.12 ± 0.01 vs. 0.14 ± 0.02, *P* < 0.05). DOX-treated mice displayed higher planar and less spherical anisotropy (*C*_Planar_ 0.07 ± 0.01 vs. 0.09 ± 0.01, *P* < 0.01; *C*_Spherical_ 0.89 ± 0.01 vs. 0.87 ± 0.02, *P* < 0.05). *C*_Planar_ and *C*_Spherical_ yielded good discriminatory power to distinguish between mice with and without AIC (c-index 0.91 and 0.84, respectively, *P* for both < 0.05). AIC is associated with regional changes in sheetlet angle but no major abnormalities of global LV microarchitecture. The geometric shape of the diffusion tensor is altered in AIC. DTI may provide a new tool for myocardial characterization in patients with AIC, which warrants future clinical studies to evaluate its diagnostic utility.

**Supplementary Information:**

The online version contains supplementary material available at 10.1007/s00395-024-01039-z.

## Introduction

Anthracyclines are highly potent anti-cancer drugs with broad application in the treatment of various solid tumors and hematological malignancies. As a major complication, however, anthracyclines are associated with a dose-dependent cardiotoxicity that predisposes to heart failure [8, 13, 20, 35, 36], a condition with a morbidity and mortality comparable to that of cancer itself [33]. Anthracycline-induced cardiotoxicity (AIC) is characterized by impaired left ventricular (LV) systolic function, which is often accompanied by a loss of myocardial mass [22, 30, 37]. While cardiac wasting is a frequent finding in patients with cancer [24, 29, 32, 34], several studies have demonstrated that anthracyclines directly promote cardiac atrophy via depletion of cardiomyocyte size and number [15, 19, 34, 37]. Reduced LV mass is a predictor of adverse cardiovascular events in AIC patients [30] and has been identified as a major cause of heart failure symptomology independent of systolic dysfunction [22, 24]. Importantly, atrophic remodeling per se can contribute to a deterioration of LV biomechanics and may thus also play a role in the complex and multifactorial pathophysiology of cancer therapy-related cardiovascular toxicity (CTR-CVT) [2, 22, 25].

In general, cardiomyopathies lead to alterations in myocardial microstructure, e.g., changes in cardiomyocyte size, myofiber tract arrangement, and extracellular matrix turnover. Diffusion tensor imaging (DTI) has emerged as a novel approach in cardiovascular magnetic resonance (CMR) for characterizing myocardial microstructure through interrogation of tissue diffusion properties and consecutive reconstruction of myofiber tracts [23]. Previous DTI studies have discovered distinct abnormalities of LV microarchitecture in a variety of pathological conditions in humans and animal models [4, 11, 17, 18, 31]. Furthermore, changes in cardiac tissue composition affect myocardial diffusion properties, which can be quantified by DTI at microscopic resolution [23]. DTI parameters have been shown to provide additional diagnostic and prognostic information indicating potential utility beyond mere pathophysiological insights [4, 11, 17, 18, 31]. While myocardial remodeling during cardiac enlargement has been extensively studied over the past decades, the impact of cardiac atrophy on microstructural features of the heart remains poorly defined. CMR is an integral part of current cardiomyopathy and cardio-oncology guideline recommendations [3, 28], but application of DTI in the context of CTR-CVT has not been reported yet.

In the present study, we comprehensively characterized an experimental model of AIC and related atrophic cardiac remodeling by high-resolution DTI. We hypothesized that (1) AIC is accompanied by changes in the three-dimensional LV microarchitecture; and (2) atrophic cardiac remodeling alters diffusion properties of the LV myocardium.

## Methods

All animal experiments were performed in accordance with the German Animal Welfare Act and Directive 2010/63/EU of the European Parliament on the protection of animals used for scientific purposes. The study was approved by local authorities (G0067/21, Landesamt für Gesundheit und Soziales Berlin, Germany).

### Animal study

Male eight-week-old C57BL/6N mice were purchased from Janvier Labs (France) and maintained under identical housing conditions (12-h dark/light cycle, standard chow diet ad libitum). AIC was induced by intraperitoneal injections of Doxorubicin (Stada, Germany; DOX) according to a standard protocol [19, 38]. Briefly, mice were injected with either 5 mg/kg body weight DOX (*n* = 16) once a week for a total of five weeks or 0.9% saline in equivalent volumes as a corresponding control (*n* = 15). Animals were weighed and examined at least twice a week. Echocardiography was performed at baseline (before initiation of treatment) and at the end of the study period. Body composition was assessed via magnetic resonance spectroscopy prior to final echocardiography. After a total of 5 weeks, mice were sacrificed via cervical dislocation under deep anesthesia with Isoflurane. Analyses were performed according to treatment group (DOX vs. control) without a prespecified definition of AIC.

### Nuclear magnetic resonance spectroscopy

Body composition was analyzed by nuclear magnetic resonance spectroscopy (Minispec LF50, Bruker, United States).

### Echocardiography

Echocardiography was performed in anesthetized mice (0.5–1.5% Isoflurane in 80% oxygen) using a 30-MHz linear frequency transducer (MX400) coupled to a Vevo 3100 Preclinical Imaging System (both FUJIFILM VisualSonics, Canada) as described previously [7].

### Necropsy and tissue preparation

Tibial length was measured for normalization of organ weights and LV mass. The right superior lung lobe was weighed immediately after removal (wet weight) and again after being dried to a constant weight (dry weight) for calculation of the wet-to-dry lung weight ratio.

A subset of whole-heart specimens was prepared for DTI (*n* = 8 per group) as described before [7]. Briefly, these hearts were arrested in diastole by retrograde cardioplegia via the ascending aorta (20 mM potassium chloride in cold phosphate-buffered saline). After perfusion fixation and storage in 4% formalin for 28 days, hearts were sent to the CMR site (Comprehensive Heart Failure Center, Wuerzburg, Germany) for consecutive scans.

Remaining heart samples (*n* = 7 and 8 per group, respectively) were processed for mass spectrometry imaging after formalin fixation without prior cardioplegia.

### DTI

Whole-heart DTI measurements were performed on a 7 T PharmaScan 70/16 (Bruker BioSpin, Germany) using an in-house built solenoid coil with 3 windings. Hearts were placed in 2 ml Eppendorf tubes whose outer diameter accurately fit within the solenoid coil. Similar to prior studies [7, 27], tubes were filled with the susceptibility matching medium Fomblin™ (Solvay Specialty Polymers, Italy) to prevent distortions at tissue-medium interfaces and to adjust the receive chain of the CMR system to signal from heart tissue. If required, hearts were fixed within the Eppendorf tubes using cellulose gauze to prevent buoyancy and potential displacement of the sample during scans. Axial, coronal, and sagittal localizers with an echo time of 3.3 ms were used to assess if excess air was present within heart cavities and whether the sample was positioned properly (long axis orthogonal to bore). DTI data was acquired for 70 slices with 150 μm isotropic resolution using a spin echo sequence with standard readout and monopolar diffusion encoding (2.5 ms gradient duration and 9.5 ms gradient separation). Three reference images (*b* = 0 s/mm^2^) were acquired, while the signal attenuation induced by the diffusion process was measured in 12 directions (*b*_max_ = 1,123 s/mm^2^). Further measurement parameters were TE/TR: 17.5/3,000 ms, field of view: 10 × 10 mm^2^, matrix size: 67 × 67. Total scan time for 10 averages of the DTI protocol was 6 h and 22 min.

For post-processing, images were denoised using overcomplete local partial component analysis. LV endocardial and epicardial contours were traced, and the myocardium was divided into 17 segments (excluding the apex for metrics based on transmural profiles). Based on this segmentation strategy, the following regions of interest were defined: anterior (segments 1, 7, and 13), septal (2, 3, 8, 9, 14), inferior (4, 10, 15), and lateral (5, 6, 11, 12, 16). Endocardial and epicardial contours were also used to assess wall thicknesses and to generate transmural profiles. LV mass was calculated by multiplying CMR-derived LV myocardial volume with the myocardial density of 1.05 g/cm^3^. Helix angle (HA), HA gradients, and absolute sheetlet angles (|E2A|) were calculated as described before [27]. The geometric shape of the diffusion tensor was determined via the three eigenvalues and parameterized using established equations [12].

Except image denoising, all post processing was done using in-house developed MATLAB (MathWorks, United States) code and DSI Studio (http://dsi-studio.labsolver.org, November 15, 2018 build). All images depicting tractography of cardiomyocyte bundles were generated using tractography algorithms and visualization tools of DSI Studio. Adjustments to interface scripts connecting MATLAB and DSI studio enabled the introduction of HA values into the DSI studio data format.

Quality control indicated incomplete diastolic arrest in a single DOX-treated heart, which was consequently excluded from study results (15/16 scans with adequate quality).

### Histopathology and immunohistochemistry

Fixed cardiac cross sections derived from the LV base, mid and apex region were embedded in paraffin and stained with Picrosirius Red (Morphisto, Germany). Collagen content was defined as the relative proportion of Picrosirius Red-positive area from total LV myocardium as assessed by a software algorithm (Aperio ImageScope and Aperio GENIE, both Leica Biosystems, Germany).

### Matrix-assisted laser desorption/ionization mass spectrometry imaging

Spatial proteomics data were acquired via matrix-assisted laser desorption/ionization mass spectrometry imaging as described before [21]. Corresponding sample preparation, data acquisition, and statistics are detailed in the Supplemental Material.

### Statistics

Data are reported as mean ± SEM or median [95% confidence interval]. Normality was assessed visually via Q–Q plots and the Shapiro–Wilk test. Group differences were compared by two-tailed unpaired Student’s *t*-test (all data with normal distribution). Body weight development was analyzed by two-way repeated measures ANOVA. Relationships between continuous variables were assessed by linear regression (with 95% confidence interval). Statistical significance was assumed at a value of *P* < 0.05. Statistical analyses were performed using GraphPad PRISM 9 (GraphPad Software, United States).

## Results

### Mice with AIC developed cachexia and cardiac atrophy

**Fig. 1:**
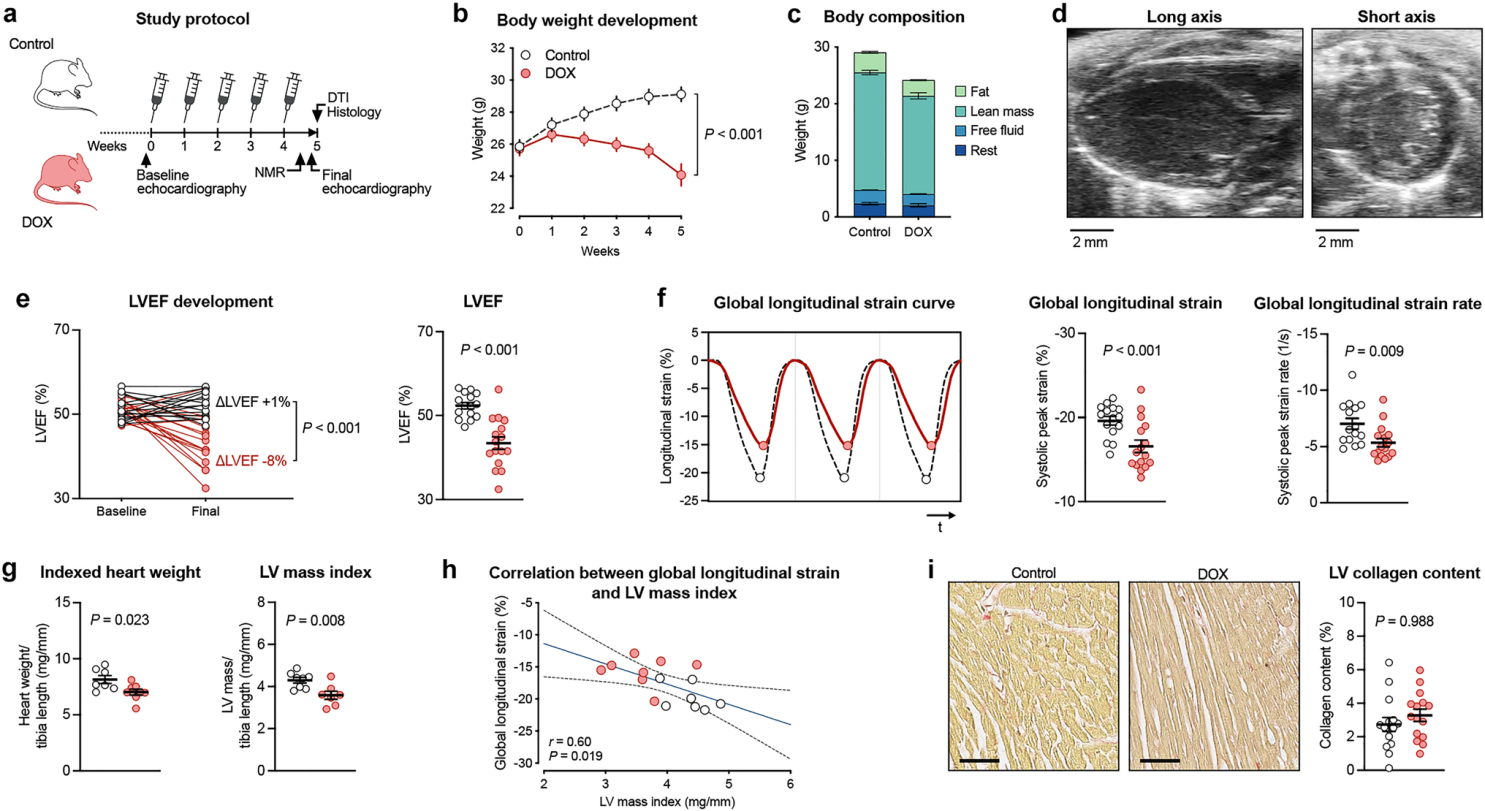
Study outline and model characterization. **a** Study protocol. **b** Body weight development during the study period. **c** Body composition by NMR at the end of the study protocol. **d** Representative echocardiographic images of the parasternal long (left) and short axis (right). **e** Individual LVEF development (left) and LVEF at the end of the study period (right). **f** Representative strain curves of global longitudinal stain during three consecutive cardiac cycles with circles representing systolic peak strain (left); analysis of global longitudinal strain (mid) and -strain rate (right). **g** Normalized heart weight during necropsy and CMR-derived LV mass index. **h** Linear correlation between global longitudinal strain and LV mass index (linear regression with 95% confidence interval). **i** Representative Picrosirius Red staining of collagen fibers in cardiac cross sections (left) and quantification of LV collagen content (right). Scale bars indicate 50 µm. *n* = 15–16 or 7–8 per group (**b**, **e**, **f**, **i** and **g**-**h**, respectively). Two-way repeated measures ANOVA (**b**), unpaired Student’s *t*-test (**e**, **f**, **g**, **i**), and linear regression analysis (**h**). *LVEF* LV ejection fraction; *NMR* nuclear magnetic resonance spectroscopy

AIC was established in mice by repetitive injections of high-dose DOX (Fig. 1a). Starting after the second injection, mice developed a progressive decline in body weight with a loss of both fat and lean mass (Fig. 1b, c). Induction of AIC was confirmed by echocardiography, where DOX-treated mice showed an impairment of LV ejection fraction and longitudinal deformation (Fig. 1d–f). Echocardiographic measurements and physiological data are summarized in Table 1. AIC was accompanied by cardiac atrophy as indicated by reduced total heart weight and lower LV mass index (Fig. 1g). Global longitudinal strain correlated significantly with LV mass index (Fig. 1h). Histopathology demonstrated no relevant myocardial fibrosis at the end of the study period (Fig. 1i).

**Table 1:** Physiological and echocardiography data at the end of the study period

	Control	DOX	*P* value
*n*	15	16	
Heart rate (1/min)	468 ± 7	479 ± 10	0.38
Lung wet/dry weight ratio	4.6 ± 0.1	4.6 ± 0.1	0.28
Conventional echocardiography			
LVEF (%)	52 ± 1	43 ± 1	**<0.001**
EDV (µl)	68 ± 4	61 ± 2	0.08
ESV (µl)	33 ± 2	34 ± 2	0.54
*E* (mm/s)	738 ± 28	605 ± 24	**0.001**
*A* (mm/s)	464 ± 29	422 ± 25	0.29
*e*′ (mm/s)	28 ± 1	27 ± 2	0.64
*A*′ (mm/s)	16 ± 2	20 ± 2	0.10
*E*/*A*	1.7 ± 0.1	1.5 ± 0.1	0.32
*E*/*e*′	26.5 ± 1.0	23.4 ± 1.5	0.10
*e*′/*a*′	26.5 ± 0.3	1.6 ± 0.2	0.13
IVRT (ms)	15.3 ± 0.5	18.1 ± 0.6	**0.002**
Speckle-tracking echocardiography			
Global longitudinal strain (%)	−19.6 ± 0.5	−16.6 ± 0.7	**0.002**
Global longitudinal strain rate (1/s)	−7.0 ± 0.5	−5.3 ± 0.4	**0.009**
Global radial strain (%)	30.1 ± 1.6	30.0 ± 1.3	0.95
Global radial strain rate (1/s)	8.0 ± 0.4	7.3 ± 0.3	0.23
Global circumferential strain (%)	−20.8 ± 0.7	−20.2 ± 0.8	0.58
Global circumferential strain rate (1/s)	−7.5 ± 0.5	−7.3 ± 0.4	0.84

Mean ± SEM; unpaired Student’s *t*-test. Bold indicates statistical significance

*A* late diastolic filling rate; *a*′ late diastolic mitral annular velocity; *E* early diastolic filling rate; *e*′ early diastolic mitral annular velocity; *EDV* end-diastolic volume; *ESV* end-systolic volume; *IVRT* isovolumic relaxation time; and *LVEF* left ventricular ejection fraction

### LV myofiber arrangement remained preserved in AIC

**Fig. 2:**
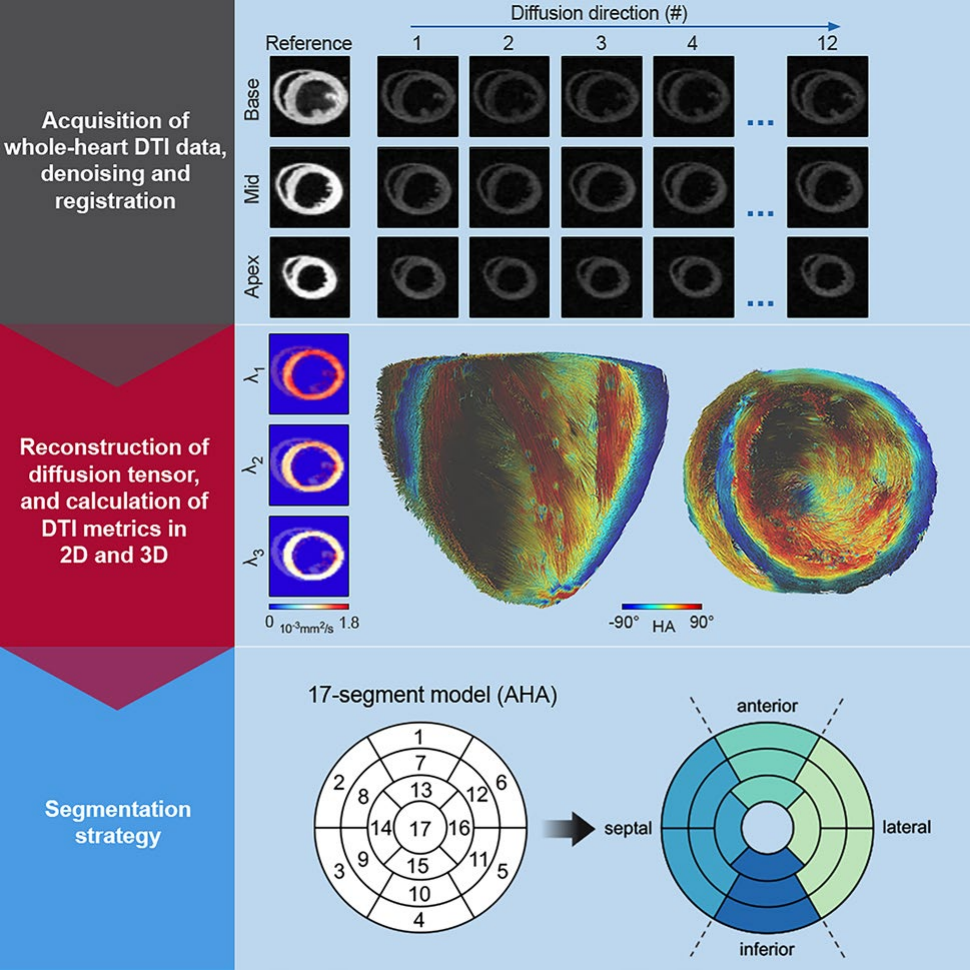
DTI workflow. Representative reference and diffusion weighted images of a DOX-treated mouse are shown for a basal, mid-cavity, and apical slice (upper panel). Reference and diffusion weighted images are displaced with consistent intensity scaling. All data were denoised using local partial component analysis. Afterwards, the diffusion tensor was calculated using DSI studio. Tensor information was imported into MATLAB to determine eigenvalues (*λ*_1,_ *λ*_2,_ *λ*_3,_ representative maps shown for the DOX-treated mouse), eigenvectors, and to calculate DTI parameters. Metrics were imported to DSI studio for tractography and whole-heart visualization (mid panel). Image analyses were performed for both the global LV myocardium and in different regions of interest using a modified segmentation strategy (lower panel). *AHA* American Heart Association

**Fig. 3:**
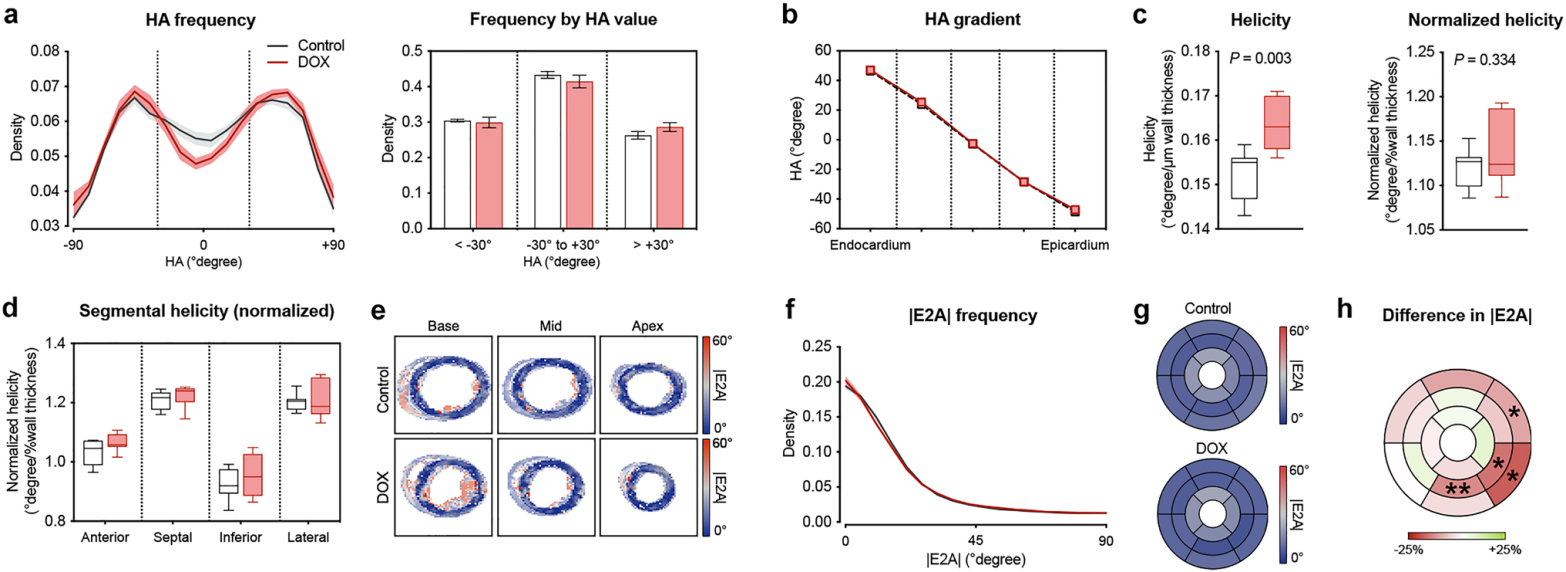
Geometric analysis of LV microarchitecture via DTI. **a** Histogram depicting frequency of fiber tracts by HA (left) and proportion of fiber tracts according to HA range (right). **b** Transmural HA gradient across the LV wall. **c** Analysis of helicity (left) and normalized helicity (right). **d** Normalized helicity in the different prespecified regions of interest. **e** Representative |E2A| maps at different levels of the left ventricle. **f** Histogram of fiber tract frequency by |E2A|. **g** Bull’s eye plot illustrating mean |E2A| values in in the two treatment groups according to myocardial segment. **h** Mean percentage difference in |E2A| between mice with and without AIC by LV segment. Asterisks indicate *P* value (*P < 0.05; **P < 0.01). *n* = 7–8 per group. Unpaired Student’s *t*-test

In a subset of hearts (*n* = 7–8 per group), geometric analysis of LV microarchitecture was performed via reconstruction of diffusion tensors and visualized using whole-heart tractography (Fig. 2). There was a non-significant shift in the proportion of voxels with a neutral HA (−30° to +30°) towards higher HA values (>30°) in mice with AIC, but no major differences in HA profiles were observed (Fig. 3a). Similarly, transmural HA gradients showed the same continuous transition of myofiber tract orientation from endocardium to epicardium in both treatment groups (Fig. 3b). Transmural helicity (HA slope) was higher owing to thinner LV walls in the AIC model (Fig. 3c), which is consistent with the lower proportion of cardiomyocyte tracts with neutral HA; however, these differences were no more evident when assessed per percent wall thickness (Fig. 3c). There were no segmental differences in helicity per percent wall thickness (Fig. 3d). Overall, absolute sheetlet angle (|E2A|) was comparable between both groups (Fig. 3e–f). On segmental level, mice with AIC displayed lower |E2A| in basal anterolateral/inferolateral as well as mid inferolateral/inferior LV segments (Fig. 3g–h).

Higher HA slope was associated with reduced LV ejection fraction and global longitudinal strain (Supplemental Table 1). No significant correlations were found between cardiac function parameters and |E2A| or helicity per percent wall thickness (Supplemental Table 1).

### Atrophic cardiac remodeling is associated with altered myocardial diffusion properties

**Fig. 4:**
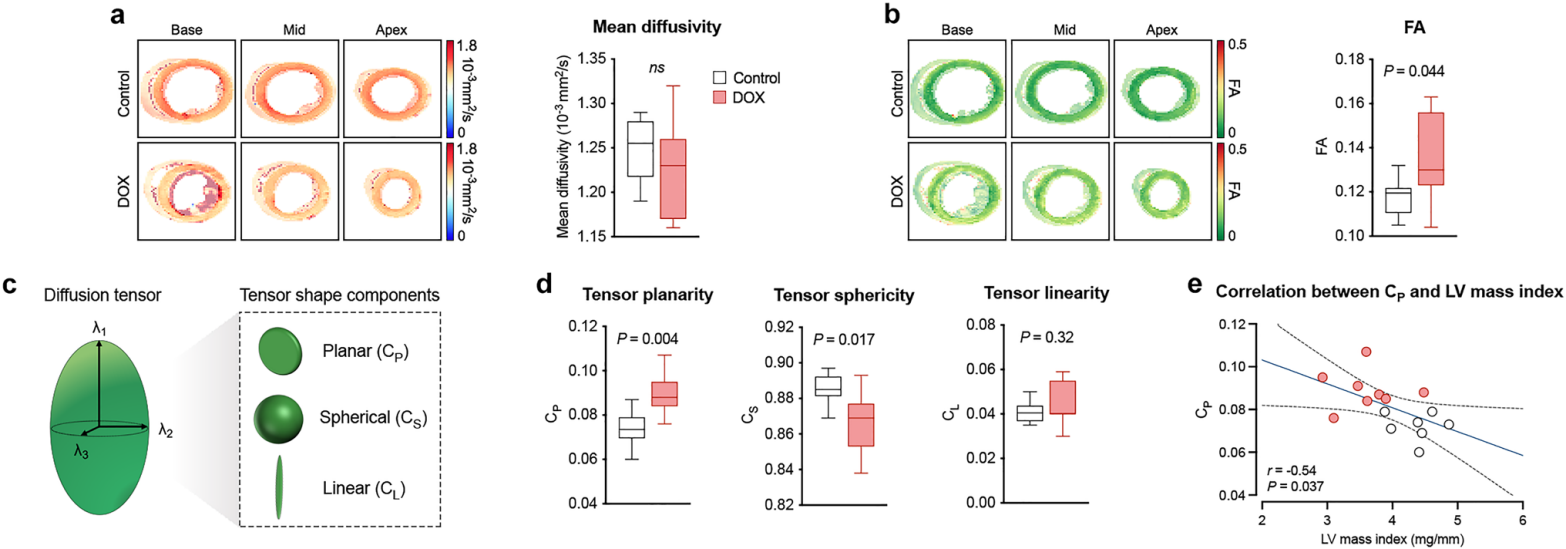
Assessment of LV diffusion properties. **a** Representative mean diffusivity maps (left) and analysis of global mean diffusivity (right). **b** Representative FA maps (left) and analysis of global FA (right). **c** Schematic illustration of diffusion tensor geometry. The relation of the three eigenvalues (*λ*_1_, *λ*_2_, and *λ*_3_) to one another determine the shape of the diffusion tensor, which can be dissected into of a planar, a spherical, and a linear component. **d** Analysis of the three geometric components of the diffusion tensor shape. **e** Correlation between tensor planarity and LV mass index (linear regression with 95% confidence interval). *n* = 7–8 per group. Unpaired Student’s *t*-test (**a**, **b**, **d**) and linear regression analysis (**e**)

The overall extent of diffusion (mean diffusivity) within the LV myocardium was similar between both experimental groups (Fig. 4a). FA, a scalar value for diffusion directionality, was higher in the AIC model (Fig. 4b). We next investigated the geometric shape of the diffusion tensor by dissecting the relations of the three eigenvalues to one another (Fig. 4c). Tensor shape was more planar and less spherical in mice with AIC (*P *for both <0.05), indicating less diffusion perpendicular to the myocyte orientation (Fig. 4d). Linear anisotropy was comparable between both groups (Fig. 4d). Tensor planarity correlated with LV mass index (Fig. 4e), whereas no significant association was observed between cardiac function parameters and global myocardial diffusion metrics (Supplemental Table 1).

### Spatial heterogeneity of myocardial adaption in response to DOX exposure

**Fig. 5:**
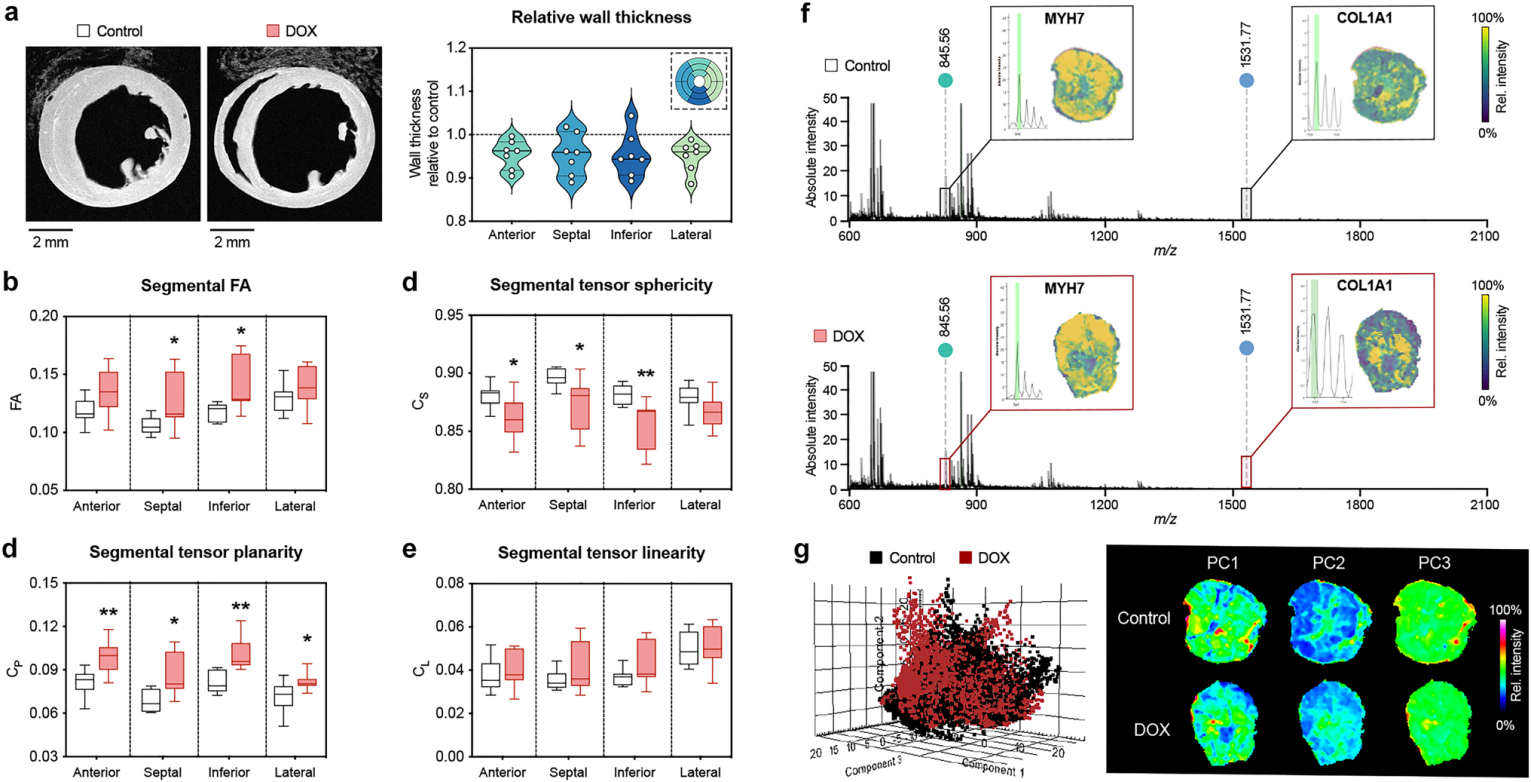
Spatial heterogeneity of myocardial adaption in AIC. **a** Representative T1 weighted images of the cardiac short axis (left) and relative wall thickness by region of interest (right). FA (**b**), tensor sphericity (**c**), tensor planarity (**d**), and tensor linearity (**e**) in the different regions of interest. **f** Representative peptide spectra throughout the left ventricle of a mouse with (upper panel) and without AIC (lower panel). Boxes show the spatial intensity distribution of peptides from MYH7 [myosin heavy chain beta] and COL1A1 [alpha-1 type I collagen] within the tissue environment of the left ventricle. **g** Principal component (PC) analysis resulted in only minor differences of the spatial peptide signatures between DOX-treated mice (red) and controls (black) (left). Representative spatial intensity distribution of the three principal components within the left ventricle showing no differences between the groups (right). *n* = 7–8 per group. Unpaired Student’s *t*-test

We next sought to evaluate regional patterns in the myocardial response to DOX exposure (Fig. 5). Wall thinning in AIC occurred without larger regional variation (Fig. 5a). FA was elevated in all regions of interest, although differences reached statistical significance at the LV septum and inferior wall only (Fig. 5b). Tensor sphericity showed a similar pattern with most pronounced alterations in septal and inferior segments (Fig. 5c). Tensor planarity was significantly higher throughout the myocardium without major regional variation (Fig. 5d). No regional differences were observed in tensor linearity (Fig. 5e). Mass spectrometry imaging showed a homogeneous spatial peptide profile throughout the LV myocardium without major group differences (Fig. 5f––g).

### Discriminatory power of DTI parameters

**Fig. 6:**
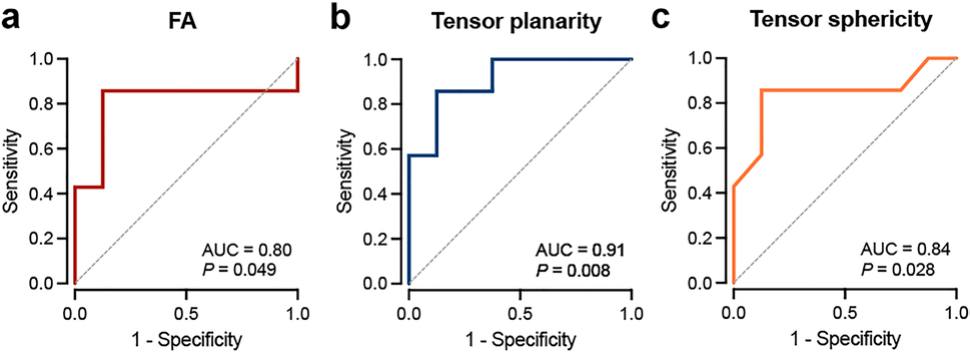
Receiver operating characteristic curves. FA (**a**), tensor planarity (**b**), and tensor sphericity (**c**) yielded good to excellent discriminatory power to distinguish mice with and without AIC. *n* = 7–8 per group

In exploratory analyses, we estimated the discrimination performance of various DTI parameters (Table 2). FA, tensor planarity, and tensor sphericity allowed to distinguish between mice with and without AIC (Fig. 6a–c); assessment of these parameters at the LV inferior wall increased sensitivity and specificity to 86 and 100%, respectively (Table 2). The discriminatory power of DTI metrics was comparable to established parameters like LV ejection fraction, global longitudinal strain, and LV mass index (Table 2).

**Table 2:** Receiver operating characteristics to discriminate between mice with and without AIC

Parameter	AUC (95% CI)	*P* value	Optimal cut off (unit)	Sensitivity (95% CI)	Specificity (95% CI)
FA	0.80 (0.54–1.00)	**0.049**	0.123 (AU)	0.86 (0.49–0.99)	0.88 (0.53–0.99)
FA inferior	0.91 (0.74–1.00)	**0.008**	1.276 (AU)	0.86 (0.49–1.00)	1.00 (0.68–1.00)
FA septal	0.79 (0.52–1.00)	0.064	0.111 (AU)	0.86 (0.49–0.99)	0.75 (0.41–0.95)
*C*_Planar_	0.91 (0.76–1.00)	**0.008**	0.082 (AU)	0.86 (0.49–0.99)	0.88 (0.53–0.99)
*C*_Planar_ inferior	0.96 (0.88–1.00)	**0.003**	0.092 (AU)	0.86 (0.49–0.99)	1.00 (0.68–1.00)
*C*_Planar_ septal	0.86 (0.66–1.00)	**0.02**	0.074 (AU)	0.86 (0.49–0.99)	0.75 (0.41–0.96)
*C*_Spherical_	0.84 (0.61–1.00)	**0.028**	0.879 (AU)	0.86 (0.49–0.99)	0.88 (0.53–0.99)
*C*_Spherical_ inferior	0.95 (0.83–1.00)	**0.004**	0.870 (AU)	0.86 (0.49–0.99)	1.00 (0.68–1.00)
*C*_Spherical_ septal	0.86 (0.64–1.00)	**0.021**	0.889 (AU)	0.86 (0.49–1.00)	0.88 (0.53–0.99)
LVEF	0.91 (0.80–1.00)	**<0.001**	47 (%)	0.75 (0.51–0.90)	1.00 (0.80–1.00)
GLS	0.81 (0.65–0.97)	**0.003**	−16.6 (%)	0.63 (0.39–0.82)	0.93 (0.70–1.00)
LV mass index	0.86 (0.64–1.00)	**0.021**	3.7 (mg/mm)	0.71 (0.36–0.95)	1.00 (0.68–1.00)

*AU* arbitrary unit; *AUC* area under the receiver operating characteristics curve; *GLS* global longitudinal strain; *LVEF* LV ejection fraction. Bold indicates statistical significance. *n* = 7–16 per group

## Discussion

**Fig. 7:**
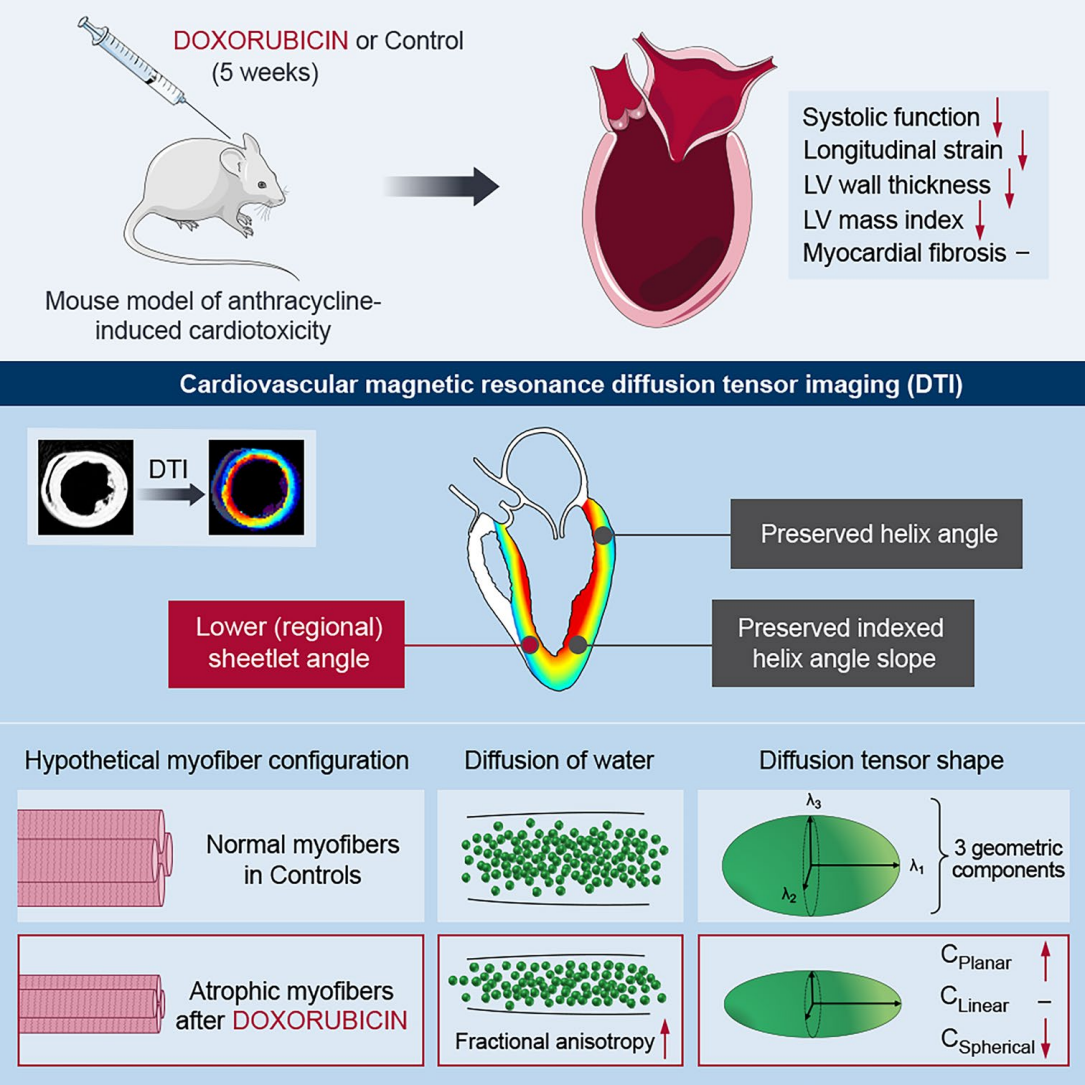
Graphical Abstract

In this first DTI study in the context of CTR-CVT and cardiac atrophy, we report three major findings (Fig. 7). First, we detected only minor alterations of the three-dimensional microfiber architecture in AIC, which were limited to few LV segments. Second, we showed changes in myocardial diffusion properties including directionality and geometric shape of the diffusion tensor in DOX-treated mice. Lastly, we identified an array of DTI parameters with potential clinical utility for diagnostic and surveillance purposes in CTR-CVT.

### Atrophic cardiac remodeling and altered microarchitecture in AIC

Cardiac atrophy and growth restriction have long been recognized in patients receiving anthracycline-based chemotherapy [25]. While reduced heart size has been postulated as a key driver of heart failure after anthracycline treatment many decades ago [25], data on the role of cardiac atrophy in CTR-CVT still remain scarce. Recent studies implicated that atrophic remodeling constitutes an early manifestation of AIC occurring within a few months after exposure to even moderate dosing regiments [22, 37]. Indeed, myocardial wall thinning in the setting of high afterload, as reported in patients treated with anthracyclines [22], may also directly promote chamber dilation and heart failure development by further increasing LV wall stress (Laplace’s law). In addition, recent data suggested that the increase in CMR-derived extracellular volume in patients with AIC may reflect shrinkage of the cellular compartment due to cardiomyocyte atrophy rather than interstitial fibrosis or edema [15]. Therefore, atrophic remodeling appears to be an important yet less perceived pathomechanism of CTR-CVT, and a better understanding of structure–function relationships in this condition is urgently needed.

Previous DTI studies have reported profound changes of global myocardial microstructure in different conditions linked to cardiac enlargement [4, 11, 17, 18, 31]. AIC and cardiac atrophy, by contrast, led to rather subtle and regional alterations of the three-dimensional myofiber arrangement in the present study. Compared to healthy controls, mice with AIC displayed a lower diastolic sheetlet angle (|E2A|) in certain segments of the left ventricle. To the best of our knowledge, reduced diastolic sheetlet angle has not been reported for other pathologies yet, whereas several studies attributed *higher* |E2A| to a more contracted and hence impaired diastolic state [17, 18, 31]. This suggests that reduced |E2A| might represent a rather specific marker of atrophic cardiac remodeling/AIC.

It appears likely that lower |E2A| reflects redistribution of sheetlets due to LV wall thinning rather than an overrelaxed diastolic state in cardiac atrophy. While anatomical assessment was limited by the small size of the murine heart, we assume that segmental differences in |E2A| may be related to regional variations in wall thickness, as observed in other conditions [16]. However, sheetlet mobility throughout the cardiac cycle as well as close examination of the segmental variation in diffusion weighted signals in AIC need to be addressed by future studies, preferably in larger animal models or human subjects.

Although cardiac atrophy occurs early even after moderate dosing regiments, the present study investigated cardiac remodeling in overt AIC. Therefore, additional studies are needed to address whether changes in LV geometry and microstructure are dose-dependent. Similarly, it remains unclear if our findings are general features of atrophic cardiac remodeling or if LV adaption varies among different etiologies of cardiac atrophy.

In contrast to findings from hypertrophic phenotypes, we observed no changes in HA or helicity, which is in line with generally preserved myocardial microstructure during atrophic remodeling and AIC.

### Impact of AIC on myocardial diffusion properties

Diffusion weighted CMR provided novel metrics for myocardial tissue characterization in AIC. We found a higher directionality and a modified geometric shape of the diffusion tensor in DOX-treated mice, which correlated with atrophic cardiac remodeling. This is in accordance with previous work from skeletal muscle tissue that showed a close relationship between myocyte size and related diffusion parameters (FA, *λ*_2_ and *λ*_3_) [6]. Smaller cell cross-sectional area leads to increased molecule-surface interactions. Diffusion displacement within that cross-sectional plane is thus reduced, which is expressed by lower secondary and tertiary eigenvalues [5]. This, in turn, translates into alterations in directionality and geometric shape of the diffusion tensor given the inherent link between these metrics [5]. Importantly, the parameters’ sensitivity has been shown to increase with decreasing myofiber size, indicating particular utility in the context of myofiber atrophy [6]. Therefore, DTI might represent a useful method for noninvasive myocardial characterization in conditions associated with atrophic cardiac remodeling like AIC. As such, increased FA and increased tensor sphericity may be quite specific diagnostic markers, given that several cardiac pathologies exhibit an opposite DTI pattern [4, 11, 17, 18]. FA, tensor planarity, and tensor sphericity yielded good to excellent discriminatory power to distinguish between mice with and without AIC in the present study, and future investigation are warranted for evaluation of the diagnostic value of DTI in the clinical setting.

In addition to cardiomyocyte size, diffusion properties are influenced by alterations of myocardial tissue composition such as extracellular matrix expansion [23]. In contrast to previous studies using the same experimental protocol [38], we observed no myocardial fibrosis in mice with AIC as measured by two different approaches. Thus, increased collagen content seems unlikely to affect myocardial diffusion properties to a relevant extent in the present investigation. However, myocardial fibrosis should be considered as a potential confounder during future DTI studies using similar experimental outlines.

### Diagnostic potential of DTI in AIC

CMR represents a cornerstone in the evaluation of patients with cardiomyopathies and CTR-CVT [1, 3, 28]. Our findings indicate that DTI may provide additional diagnostic information in CTR-CVT and cardiac atrophy via interrogation of altered diffusion properties. We used a standard mouse model of overt AIC with features of patients receiving high-dose anthracycline therapy (systolic dysfunction, cardiac atrophy, systemic side effects) [19, 38]. In this setting, diffusion metrics showed a similar discriminatory performance compared to that of cardiac function parameters. Future studies should evaluate if changes in myocardial diffusion properties precipitate overt AIC and if these measures may outperform currently used parameters during early cardiotoxicity.

Different CMR approaches for the noninvasive monitoring of cardiomyocyte size have been proposed [5, 9, 10, 14, 15]. The present study highlights the potential ability of DTI to infer atrophic cardiac remodeling, thereby validating and corroborating previous results in cardiomyocyte hypertrophy [5, 9]. With the advent of clinical DTI [23], this experimental work may serve as a pilot for future studies in patients with CTR-CVT and cardiac atrophy. Our data indicate considerable regional variation in the myocardial response to anthracycline exposure, which awaits further investigation in clinical trials.

### Study limitations

Although we used a well-characterized standard mouse model of CTR-CVT, it may not accurately reflect the multimorbidity and chronic condition of patients with cancer. While the model is considered to mimic chronic CTR-CVT, differences in the disease course (5 weeks versus months/years in patients) should be acknowledged. The intraperitoneal route of DOX-administration and potential peritoneal injury could have contributed to the cachectic phenotype, which further limits its comparability. Only a relatively small sample size has been studied by DTI owing to long acquisition times and complex image post-processing. DTI has been performed ex vivo to achieve whole-heart coverage and images at the highest possible resolution, which limits data to the end-diastolic phase. While fixation time was identical for all samples to control for the impact of formalin on diffusion properties and tissue shrinkage [26], these aspects should be considered during data interpretation (e.g., comparisons with other studies using different tissue processing). Histological validation of cardiomyocyte atrophy was not feasible due to the long fixation with formalin and needs to be addressed by future studies.

## Conclusions

In an experimental model of AIC, we identified distinct alterations of myocardial diffusion properties linked to atrophic cardiac remodeling. The three-dimensional myofiber architecture remained largely preserved in AIC. Based on the findings of this proof-of-concept study, DTI may provide a new set of diagnostic parameters to noninvasively monitor the myocardial response to anthracycline exposure. Future studies are warranted to evaluate the clinical utility of DTI in patients with CTR-CVT and other conditions associated with cardiac atrophy.

## Supplementary Information

Below is the link to the electronic supplementary material.

Supplementary file1 (PDF 153 KB)

## Data Availability

The authors declare that the data supporting the findings of this study are available within the article, and in the supplemental material.

## Abbreviations

AIC: Anthracycline-induced cardiotoxicity
CMR: Cardiovascular magnetic resonance
CTR-CVT: Cancer therapy-related cardiovascular toxicity
DOX: Doxorubicin
DTI: Diffusion tensor imaging
FA: Fractional anisotropy
HA: Helix angle
LV: Left ventricular
