# Supplementary material for: Characterization of anthracycline-induced cardiotoxicity by diffusion tensor magnetic resonance imaging
Source: Basic Res Cardiol. 2024 Mar 14;120(1):57–69. doi: 10.1007/s00395-024-01039-z (PMC11790774; doi:10.1007/s00395-024-01039-z)
Supplement: Supplementary file 1 — Supplementary file1 (PDF 153 KB) [file 395_2024_1039_MOESM1_ESM.pdf]

SUPPLEMENTAL MATERIAL

Supplemental methods

Matrix-assisted laser desorption/ionization mass spectrometry imaging (MALDI-MSI)

6-µm sections from paraffin-embedded cardiac cross-sections were mounted onto conductive indium tin oxide coated slides (Bruker Daltonik, Germany) and dewaxed using ethanol. Following heat-induced antigen retrieval and trypsin digestion, matrix solution (α-cyano-4-hydroxycinnamic acid) was applied with an automated spraying device (HTX TM-Sprayer, HTX Technologies, United States).

MALDI-MSI data were acquired on a rapifleX® MALDI Tissuetyper system with flexControl 3.0 and flexImaging 3.0 (all Bruker Daltonik, Germany) using the following settings: reflector mode, mass detection range *m/z* 800-3200, 500 laser shots per spot, sampling rate 1.25 GS/s, raster grid width 50 µm. External calibration was performed using a peptide calibration standard (Bruker Daltonik). Potential contaminations were assessed for each slide outside the tissue section. After MALDI-MSI experiments, matrix was removed with ethanol, and tissue sections were stained with hematoxylin/eosin for histology. MALDI-MSI raw data were evaluated with SCiLS Lab software (Version2021b, SCiLS GmbH, Germany). Data pre-processing included convolution baseline removal (width: 20) and total ion count normalization. The orthogonal matching pursuit algorithm was used for the selection of peaks, and top-down segmentations were performed by bisecting k-means clustering, ±0.156 Da interval width, mean interval processing and medium smoothing strength. Receiver operating characteristic analyses were performed to detect characteristic peptide *m/z* values to distinguish mice with and without AIC. Peptide values (*m/z* peaks) with an AUC < 0.4 or > 0.6 were subsequently analysed using the Wilcoxon rank sum test. Statistical significance was assumed

at a *P* value < 0.001. Supervised principal component analysis was conducted to define characteristic peptide signatures that allow differentiation between both experimental groups. Protein identification was performed in adjacent tissue sections using liquid chromatography electrospray ionization tandem mass spectrometry (Dionex UltiMate 3000, Thermo Fisher Scientific, United States) coupled to an ESI-QTOF ultrahigh-resolution mass spectrometer (Impact II, Bruker Daltonic). Similar to their preparation for MALDI-MSI, sections were preheated to 80 °C for 15 min before deparaffinization. Paraffin removal, antigen retrieval and tryptic digest were carried out as for MALDI-MSI. After incubation at 50 °C in a humidity chamber saturated with potassium sulfate solution for 2 h, peptides were extracted from tissue section into 40 µL of 0.1% trifluoroacetic acid and incubated for 15 min at room temperature. Digests were filtered using a ZipTip® C18 following the manufacturer's instructions, and the eluates were vacuum concentrated (Eppendorf® Concentrator 5301, Eppendorf AG, Hamburg, Germany) and reconstituted separately in 20 µL 0.1% trifluoroacetic acid, from which 2 µL were injected into a NanoHPLC (Dionex UltiMate 3000, Thermo Fisher Scientific) coupled to an ESI-QTOF ultrahigh-resolution mass spectrometer (Impact II™, Bruker Daltonic GmbH, Bremen, Germany). For identification, PEAKS X Pro (Version Peaks Studio 10.6) SW were used followed by database analysis using the following parameters: De novo sequencing: Parent Mass Error Tolerance 20.0 ppm, Fragment Mass Error Tolerance 0.05 Da, Enzyme Trypsin, Variable Modifications Oxidation (M), Deamidation (NQ) and Acetylation (N-term), Max Variable PTM Per Peptide 3. Database Search: Search Engine Name PEAKS, Parent Mass Error Tolerance 20.0 ppm, Fragment Mass Error Tolerance 0.05 Da, Precursor Mass Search Type monoisotopic, Enzyme Trypsin, Max Missed Cleavages 3, Digest Mode Specific, Variable Modifications Oxidation (M), Deamidation (NQ) and Acetylation (N-term), Max Variable PTM Per Peptide 3,Database UniProt_SwissProt, Taxonomy Mus musculus. A peptide

significance filter -lgP >20, a protein significance filter -lgP >15 and unique-peptide filter was set to 1.

Briefly, the comparison of MALDI-MSI and LC-MS/MS m/z values required the identification of >1 peptide (search mass window < 0.3 Da). Only peptides with the smallest mass differences in the mass window and a correlation ratio ≥0.30 were counted as a match. The peptides with highest MOWSE peptide scores and the smallest mass differences between MALDI-MSI and LC-MS/MS data were accepted as correctly identified.

Supplemental Tables

Supplemental Table 1: Relationship between DTI metrics and cardiac function. LVEF, LV ejection fraction; *r*, Pearson's correlation coefficient. Bold indicates statistical significance. n=15

	**Global longitudinal strain**		**LVEF**	
**Parameter**	***r***	***P* value**	***r***	***P* value**
FA	0.39	0.15	0.14	0.61
FA inferior	0.46	0.085	0.10	0.72
FA septal	0.41	0.13	0.10	0.72
C_{Planar}	0.44	0.10	-0.08	0.78
C_{Planar} inferior	0.52	**0.047**	-0.04	0.88
C_{Planar} septal	0.43	0.11	-0.05	0.86
$C_{Spherical}$	-0.43	0.11	-0.07	0.80
$C_{Spherical}$ inferior	-0.49	0.064	-0.06	0.84
$C_{Spherical}$ septal	-0.42	0.12	-0.06	0.85
|E2A|	-0.11	0.71	0.12	0.67
|E2A| inferior	-0.25	0.38	0.28	0.31
|E2A| lateral	-0.18	0.52	0.23	0.42
Helicity (°degree/µm)	0.56	**0.030**	-0.46	0.081
Normalized helicity (°degree / %wall thickness)	0.38	0.17	-0.27	0.33
